# Progress and challenges in integrating CVCT into routine antenatal services in government clinics in Ndola, Zambia

**DOI:** 10.1186/1742-4690-9-S2-P217

**Published:** 2012-09-13

**Authors:** N Czaicki, J Davitte, B Siangonya, I Thior, J Pulerwitz, M Inambao, S Allen

**Affiliations:** 1 Emory University, Atlanta, GA, USA; 2 Rwanda Zambia HIV Research Group, Ndola, Zambia; 3 PATH, Washington, DC, USA

## Background

In 2008, the Ministry of Health in Zambia recommended that HIV testing be provided to partners of antenatal clients. Herein the Zambia Emory HIV Research Project (ZEHRP) in collaboration with the Arise Program/PATH funded by CIDA examines the transition of Couples’ Voluntary HIV Counseling and Testing (CVCT) from NGO-sponsored weekend services to integrated weekday services in government clinics in Ndola, the second largest city in Zambia. We also describe how CVCT data are being recorded and reported.

## Methods

Data were extracted from government-issued logbooks in antenatal clinic (ANC), prevention of mother to child (PMTCT), and voluntary counseling and testing (VCT) services in the six largest government clinics for the year 2010, and in15 clinics in the first half of 2011. CVCT procedures and record-keeping were documented through observation and counselor interviews.

## Results

In 2010, only one of the six largest clinics tested more than four couples/week. In this clinic, a community promotions campaign in Q2 resulted in an average of 5 couples/day seeking testing, though numbers tapered off over time to 2 couples/day by Q4. In March-May of 2011, 11 of 15 clinics averaged less than 10 couples per month. Four clinics recorded 20 to 60 couples/month: 77% of couples were seen in ANC with the remainder tested in VCT. Obstacles included low participation of men, lack of staff trained to counsel couples jointly, procurement challenges for HIV tests for men in ANC, and non-uniform recording of CVCT in ANC and VCT logbooks.

## Conclusion

This study identified several challenges for integrating CVCT into regular clinic services. To address these challenges, we recommend implementing new data recording instruments, increasing training of counselors and nurses in CVCT, prioritizing ANC clients attending with partners and expanding of community sensitization using proven models.

